# Immunological Value of Prognostic Signature Based on Cancer Stem Cell Characteristics in Hepatocellular Carcinoma

**DOI:** 10.3389/fcell.2021.710207

**Published:** 2021-08-02

**Authors:** Qianhui Xu, Hao Xu, Shaohuai Chen, Wen Huang

**Affiliations:** ^1^The Second Affiliated Hospital and Yuying Children’s Hospital of Wenzhou Medical University, Wenzhou, China; ^2^Department of Hepatobiliary and Pancreatic Surgery, The Second Affiliated Hospital, School of Medicine, Zhejiang University School of Medicine, Hangzhou, China

**Keywords:** liver cancer stem cells, hepatocellular carcinoma, tumor immune microenvironment, immunotherapy, prognosis

## Abstract

**Background:** Liver cancer stem cells, characterized by self-renewal and initiating cancer cells, were decisive drivers of progression and therapeutic resistance in hepatocellular carcinoma (HCC). However, a comprehensive understanding of HCC stemness has not been identified.

**Methods:** RNA sequencing information, corresponding clinical annotation, and mutation data of HCC were downloaded from The Cancer Genome Atlas-LIHC project. Two stemness indices, mRNA expression-based stemness index (mRNAsi) and epigenetically regulated-mRNAsi, were used to comprehensively analyze HCC stemness. Estimation of Stromal and Immune Cells in Malignant Tumors using Expression Data and single-sample gene-set enrichment analysis algorithm were performed to characterize the context of tumor immune microenvironment (TIME). Next, differentially expressed gene (DEG) analysis and weighted gene co-expression network analysis (WGCNA) were employed to identify significant mRNAsi-related modules with hub genes. Kyoto Encyclopedia of Genes and Genomes and Gene Ontology enrichment pathways were analyzed to functionally annotate these key genes. The least absolute shrinkage and selection operator (LASSO) Cox regression analysis was performed to establish a prognostic signature. Kaplan–Meier survival curves and receiver operating characteristic (ROC) analysis were applied for prognostic value validation. Seven algorithms (XCELL, TIMER, QUANTISEQ, MCPcounter, EPIC, CIBERSORT, and CIBERSORT-ABS) were utilized to draw the landscape of TIME. Finally, the mutation data were analyzed by employing “maftools” package.

**Results:** mRNAsi was significantly elevated in HCC samples. mRNAsi escalated as tumor grade increased, with poor prognosis presenting the higher stemness index. The stemness-related (greenyellow) modules with 175 hub genes were screened based on DEGs and WGCNA. A prognostic signature was established using LASSO analysis of prognostic hub genes to classify samples into two risk subgroups, which exhibited good prognostic performance. Additionally, prognostic risk-clinical nomogram was drawn to estimate risk quantitatively. Moreover, risk score was significantly associated with contexture of TIME and immunotherapeutic targets. Finally, potential interaction between risk score with tumor mutation burden (TMB) was elucidated.

**Conclusion:** This work comprehensively elucidated that stemness characteristics served as a crucial player in clinical outcome, complexity of TIME, and immunotherapeutic prediction from both mRNAsi and mRNA level. Quantitative identification of stemness characteristics in individual tumor will contribute into predicting clinical outcome, mapping landscape of TIME further optimizing precision immunotherapy.

## Introduction

As specific cell types of cancer cell population with stem-like properties to self-renewal and promoting cancer cell proliferation and invasion, cancer stem cells (CSCs) caused the heterogeneous cancer cell lineages (De Francesco et al., 2018). Additionally, it was well established that activation of CSCs was the main driver of tumorigenicity, progression, and chemotherapy resistance (Vidal et al., 2014; Clarke, 2019; Xu et al., 2021a). Besides, one-class logistic regression (OCLR)-based transcriptomic and epigenetic signatures were created and employed to compute the stemness index, with the mRNA expression-based stemness index (mRNAsi) reflecting gene expression, and the epigenetically regulated (EREG)-mRNAsi reflecting epigenetically regulated mRNAsi (Malta et al., 2018).

Primary liver cancer is one of the most common cancers characteristic with high mortality globally (Bray et al., 2018; Forner et al., 2018; Yang et al., 2019). Based on histological stratification, 80% of liver cancer cases can be classified into hepatocellular carcinoma (HCC) (Bray et al., 2018). Such pathogenic factors for HCC such as alcohol abuse, infections of hepatitis virus, type 2 diabetes, aflatoxin exposure, and obesity served as pivotal players in hepatocarcinogenesis (Yang et al., 2019). Additionally, HCC was regarded as malignant disease experienced complicated molecular events from genomics and genetic standpoint, leading to high heterogeneity both in intertumoral and intratumor levels (Schulze et al., 2016; Cancer Genome Atlas Research Network, 2017; Liu et al., 2018; Woo and Kim, 2018). Besides, tumor–node–metastasis (TNM) staging classification was applied in clinical practice but was limited in predicting clinical outcome because etiologies of HCC diverse well among distinct population (Edge and Compton, 2010; Marano et al., 2015). It is necessary, therefore, to identify powerful tools for predicting prognosis and estimating clinical outcome, further optimizing precision clinical intervention.

Cancer immunotherapy harnessed an antitumor immune response to recognize, then eliminate, the tumor cells through activating the host’s immune system (Brahmer et al., 2015; Weber et al., 2015; Cella et al., 2016; Reck et al., 2018). Immune checkpoint blockade treatment (i.e., anti-PD-1, etc.) have dramatically made breakthrough in a great body of malignancies; however, clinical trials of anti-PD-L1 antibodies and CTLA-4 antibodies have been mostly disappointing in HCC (El-Khoueiry et al., 2017; Xu et al., 2021b). A primary reason for limited therapeutic efficacy likely lies in an extremely immunosuppressive tumor microenvironment (Nishida and Kudo, 2017). Accounting for approximately 50% of the tumor cellular population, infiltrating immune cells mostly served as opposing roles in anti-tumor response (Ringelhan et al., 2018; Xu et al., 2021c). In the recent years, mounting evidence supported that CSCs play a decisive role in the diversity of tumor immune microenvironment (TIME) and might be a key to unlocking a new era of antitumor treatments. Previous literatures reported that CSCs were significantly correlated with the development of HCC (Wu et al., 2020); however, a comprehensive analysis of HCC stemness was still lacking.

Herein, this study was designed to perform a systematic investigation of multi-omics data to identify the prognostic value and therapeutic significance of the HCC stemness *via* bioinformatic analysis. Data of RNA-seq and clinical information of HCC samples were obtained from The Cancer Genome Atlas (TCGA) portal. The interaction was first explored of mRNAsi with clinical variables and designated immunotherapeutic markers. A preliminary association between HCC stemness and immune infiltration was performed under single-sample gene-set enrichment analysis (ssGSEA) approach and ESTIMATE algorithm. Then, the differentially expressed genes (DEGs) were recognized followed by weighted gene co-expression network analysis (WGCNA), in which 175 stemness-related genes (greenyellow module) were determined. Next, the candidate genes in the module were further screened using univariate COX regression analysis and least absolute shrinkage and selection operator (LASSO) regression analysis. Then, a multi-gene prognostic risk model and a risk-clinical nomogram were established. The prognostic value was validated using multiple methods and an external testing group (ICGC-LIRI-JP). Additionally, the potential role of risk signature in TIME and immunotherapy was explored. Finally, the synergistic effect of risk score with gene mutation was demonstrated. These findings may contribute a novel insight into potential targets and advance precision immunotherapy for HCC.

## Materials and Methods

### Public Data Acquisition

The RNA-seq profile of 50 normal liver and 374 HCC samples (377 samples excluding 3 replicated samples sharing the same origin with other samples) with corresponding clinical data were obtained from TCGA^1^ database. The GTF annotation file was downloaded from the Ensembl Genome Browser^2^ to convert the Ensembl gene ID into the gene symbol and extract the mRNA profile.

The stem cell indices based on the transcriptome of each normal liver and HCC specimen were obtained from a previously reported study (Malta et al., 2018), and referred to as the mRNAsi and the EREG-mRNAsi in the following sections (Supplementary Table 1). The Stemness Index Workflow was described as follows^3^ : the gene names were first mapped from Ensembl IDs to Human Genome Organization, dropping any genes that had no such mapping. The data were centered, then OCLR was applied to just the samples labeled SC (which included both ESC and iPSC). Once the signature is obtained, it can be applied to score new samples. Spearman correlations were computed between the model’s weight vector and the new sample’s expression profile. The approach was validated using leave-one-out cross-validation by withholding each SC sample in turn. A separate signature was then trained on all other SC samples and used to score the withheld sample as well as all the non-SC samples. Having validated the signature by using cross-validation and external SC data, it was then applied to score the TCGA PanCancer cohort using the same Spearman correlation (RNA expression) operators. The indices were subsequently mapped to the (0,1) range by using a linear transformation that subtracted the minimum and divided by the maximum (Malta et al., 2018). Four categories of somatic mutation data of HCC patients were obtained from TCGA portal. We singled out the mutation files, which were obtained through the “SomaticSniper variant aggregation and masking” platform for subsequent analysis. The expression profiling information and corresponding clinical data were downloaded from the ICGC.^4^ The detailed clinical data of HCC patients from TCGA-LIHC and ICGC-LIRI-JP are recorded in Supplementary Table 2.

### Associations Between the Stemness Index and Clinicopathological Variables

Hepatocellular carcinoma samples were employed for subsequent analysis except sample without mRNAsi or clinical data. The mRNAsi was defined as an index between 0 and 1, which could estimate the activity of CSC and HCC samples, which were divided into low- and high-mRNAsi subgroups after the median mRNAsi score (0.3845) was set as the cut-off point. In addition to the mRNAsi, the EREG-mRNAsi, of which the median value was 0.5895, was also selected as the stemness index. Kaplan–Meier (K–M) curve was then analyzed to compare the overall survival (OS) between the two subgroups with the log-rank test. The comparison of mRNAsi between subgroups under clinical features was performed with the “limma” and “ggpubr” package in R.

### Identification of Differentially Expressed Genes

The “limma” R package with false discovery rate (FDR) < 0.05 and |log2 fold change| > 1 was employed to recognize DEGs between normal and HCC samples. DEGs meeting the selection criteria were extracted for further research. Volcano and heatmap plot were drawn using the “limma” and “pheatmap” packages, respectively.

### Correlation Between Stemness and Immune Infiltration

The Estimation of Stromal and Immune Cells in Malignant Tumors using Expression Data (ESTIMATE) algorithm (Yoshihara et al., 2013), as a new algorithm based on the unique properties of the transcriptional profiles, could estimate the tumor cellularity and the tumor purity.

The immune score and stromal score were calculated to quantify the relative enrichment of immune and stromal cells, which form the basis for the ESTIMATE score to predict tumor purity. The correlation was then analyzed between mRNAsi and these scores or tumor purity. The *p*-values of the relevance were calculated using the Spearman test.

### Single-Sample Gene-Set Enrichment Analysis

Single-sample gene-set enrichment analysis algorithm was used to infer the level of infiltrating immune cells with the gene sets (Hänzelmann et al., 2013). The ssGSEA value was normalized to a percentile distribution, where 0 was the minimum value of immune cell abundance, and 1 was the maximum score. The R package “GSEABase” with 29 immunity-related signatures was employed to predict the relative enrichment of antitumor immunity.

### Weighted Gene Co-expression Network Analysis

The gene-expression profiles with a total of 7,667 DEGs identified previously were applied to explore the mRNAsi-related modules using R package “WGCNA” (Langfelder and Horvath, 2008). Data RNA-seq was first filtered to reduce outliers. The co-expression similarity matrix consisted of the absolute values of the correlation between transcript expression levels. A Pearson correlation matrix was constructed for paired genes. A weighted adjacency matrix was constructed using the power function amn = | cmn| β (cmn = Pearson correlation between gene m and gene n; amn = adjacency between gene m and gene n). The parameter β emphasized a strong correlation between genes and penalized a weak correlation. Next, an appropriate β value was selected to increase the similarity matrix and achieve a scale-free co-expression network. The adjacency matrix was then converted into a topological overlap matrix (TOM), which measures the network connectivity of genes defined as the sum of adjacent genes generated by all other networks. Average linkage hierarchical clustering was performed based on TOM-based dissimilarity measurements, and the minimum size (genome) of the gene dendrogram was 50. Then, module significance (MS) was calculated and used to estimate the correlations of an mRNAsi value with the different modules and record the genes in each module. The genes in each module were considered module eigengenes (MEs). The correlations between an mRNAsi value and genes were measured by gene significance (GS). Module membership (MM) was defined as the correlation between a DEG expression profile and the module genes. In addition to the mRNAsi, the EREG-mRNAsi was also selected as the clinical phenotype. Finally, genes in the module most significantly correlated with clinical traits were extracted for subsequent analysis.

### Functional Annotation

Taking advantage of R package “org.Hs.eg.db,” the Entrez ID for each mRNAsi-related gene was obtained. To elucidate underlying mechanisms of the hub genes associated with mRNAsi in biological process, we implemented the Kyoto Encyclopedia of Genes and Genomes (KEGG) and Gene ontology (GO) pathways annotation with “clusterProfiler,” “enrichplot,” and “ggplot2” packages and visualized the results.

### Establishment of mRNAsi-Related Multi-Gene Prognostic Signature

Hepatocellular carcinoma patients with missing clinical information or expression data were excluded in order to reduce statistical bias in our analysis. Univariate Cox analysis of OS was first implemented to identify mRNAsi-related genes with prognostic significance (*p* < 0.005). To avoid the overfitting of risk, the LASSO regression analysis was performed using the “glment” R package to eliminate the highly correlated genes and develop a prognostic signature (Tibshirani, 1997; Simon et al., 2011). The independent variable in the regression was the normalized expression matrix of candidate prognostic mRNAsi-related genes, and the response variables were OS and survival status of patients. The risk scores of the patients were calculated according to the normalized expression level of each gene and its corresponding regression coefficients. The formula was established as follows: score = esum (each gene’s expression × corresponding coefficient). Finally, prognostic risk model including six hub mRNAsi-related genes was constructed, and the risk score was calculated as the formula: risk score = βgene 1 × expression level of gene 1 + βgene 2 × expression level of gene 2 + …… + βgene n × expression level of gene n. Here, β was the regression coefficient in the LASSO Cox regression analysis. HCC samples were stratified into low- and high-risk subgroups when setting the median value of risk score as the cut-off point.

### Validation of the Multi-Gene Prognostic Signature

First, K–M survival analyses were performed with “survival” R package. Subsequently, the receiver operating characteristic (ROC) curves were plotted to estimate the prognostic value. Furthermore, univariate and multivariate Cox regressions were employed for prognostic validity of risk score as an independent indicator. The ICGC-LIRI-JP cohort with 231 HCC patients was used as an independent validation group and classified into high- and low-risk subgroups according to the median threshold of the TCGA dataset. The prognostic predictive precision was further validated in the external validation group.

### Risk Score With Clinical Features

To elucidate the clinical significance of risk score, the correlation analysis between risk score with such main clinicopathological variables as gender, age, pathological staging, and TNM categories was performed. To visualize the correlation of risk score with clinicopathological variables, R “pheatmap” package was employed and compared clinical characteristics between low- and high-risk patients.

### Risk Score With Tumor Immune Microenvironment Characterization

To uncover the correlation between the risk score and tumor-infiltrating immune cells, we implemented the seven algorithms including XCELL, TIMER, QUANTISEQ, MCPcounter, EPIC, CIBERSORT, and CIBERSORT-ABS to quantify the immune-infiltrating situation. Spearman correlation was analyzed to explore the relevance between risk score and the immune infiltration statues. We compared the differences in immune-infiltrating cell fraction between low- and high-risk subgroups.

### Role of Risk Score in Immune Checkpoint Blockade Treatment

According to previous research, expression patterns of immune checkpoint blockade (ICB)-related hub targets might contribute into efficacy of immunotherapy administration (Goodman et al., 2017). In this study, we fetched six hub genes of immunotherapy: programmed death ligand 1 (PD-L1, also known as CD274), programmed death 1 (PD-1, also known as PDCD1), programmed death ligand 2 (PD-L2, also known as PDCD1LG2), cytotoxic T-lymphocyte-associated antigen 4 (CTLA-4), T-cell immunoglobulin domain, and mucin domain-containing molecule-3 (TIM-3, also known as HAVCR2), and indoleamine 2,3-dioxygenase 1 (IDO1) in HCC (Kim et al., 2017; Nishino et al., 2017; Zhai et al., 2018). To further explore the potential role of risk signature in immunotherapy, the correlation of prognostic signature with expression value of six ICB hub genes was analyzed. To reveal the potential role of risk score in response to immunotherapy, we systematically fetched the expression value of 47 ICB-related hub targets (i.e., PDCD1, etc.).

### Preprocess of Epigenetic Mutation Data

Tumor mutation burden (TMB) was defined as the number of somatic, coding, base replacement, and insert-deletion mutations per megabase of the genome examined using non-synonymous and code-shifting indels under a 5% detection limit. TMB value >3 was considered as high TMB. HCC patients with missing clinical information, expression data, or TMB values were excluded in order to reduce statistical bias in our analysis. The “maftools” R package was employed to detect the number of somatic non-synonymous point mutations within each sample. The somatic alterations in HCC driver genes were revealed for samples with low-/high-risk scores.

### Depiction of Prognostic Nomogram

To comprehensively estimate prognostic ability of risk score, TMB, clinical stage, gender, age, and tumor grade for 1-, 2-, and 3-year OS, time-dependent ROC curves were performed to compute the area under the curve (AUC) values (Blanche et al., 2013). To construct a quantitative risk model to predict OS rate, a nomogram including risk score and other clinical variables to predict 1/2/3-OS probability was constructed. Subsequently, the calibration curve that showed the prognostic value of as-constructed nomogram was developed.

### Statistical Analysis

Wilcoxon rank-sum test was a non-parametric statistical hypothesis test mainly used for comparisons between two groups, and Kruskal–Wallis test was suitable for two or more categories. Overall survival (OS) refers to the interval from the date of diagnosis to the date of death. Survival curves were plotted *via* the K–M log rank test. Univariate and multivariate analyses were performed *via* Cox regression models to validate the independent prognosis predictive performance of risk signature. The prognostic value for 1-, 2-, and 3-year OS was assessed with the ROC curves. A value of *p* < 0.05 was deemed statistically significant. R software (version 4.0.4) was utilized for all statistical analyses.

## Results

### Stemness Characteristics in Hepatocellular Carcinoma

**FIGURE 1:**
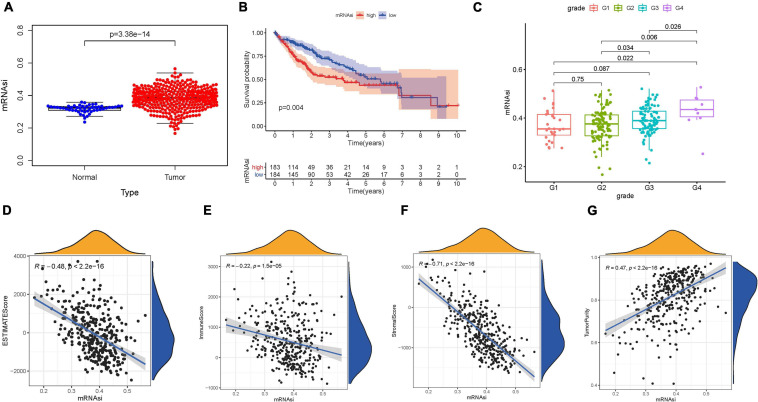
Clinical characteristics and molecular features correlated with the mRNA expression-based stemness index (mRNAsi) in hepatocellular carcinoma (HCC). **(A)** Differences in mRNAsi between normal (50 samples) and tumor (374 samples) tissues. **(B)** Kaplan–Meier curves show that the low mRNAsi subgroup had greater mortality than does the high mRNAsi subgroup. **(C)** Boxplots of mRNAsi value in individual samples stratified by tumor grade. Correlation between mRNAsi score and Estimation of Stromal and Immune Cells in Malignant Tumors Using Expression Data (ESTIMATE) score **(D)**, stromal score **(E)**, immune score **(F)**, and tumor purity **(G)**.

mRNA expression-based stemness index, as an indicator for identifying CSCs, has been used effectively to estimate the degree of differentiation of tumor cells. A significantly higher mRNAsi score was recorded in HCC tissues compared with normal samples (Figure 1A). Likewise, normal samples had significantly lower EREG-mRNAsi values than HCC samples (Supplementary Figure 1A). To further reveal the potential role of the stemness index in prognosis, K–M survival analysis was performed and indicated that lower mRNAsi value suggested longer OS times (*p* = 0.004; Figure 1B). However, there was no significant prognosis difference between low-EREG-mRNAsi and high-EREG-mRNAsi (*p* = 0.215; Supplementary Figure 1B). To elucidate the correlation of stemness with clinical variables, differential analysis was performed. For early grade and advanced grade, mRNAsi score showed a significant higher trend in advanced grade (Figure 1C). Notably, female samples experienced lower EREG-mRNAsi scores relative to male samples (Supplementary Figure 1C).

### Association of the Stemness With Context of Tumor Immune Microenvironment

**FIGURE 2:**
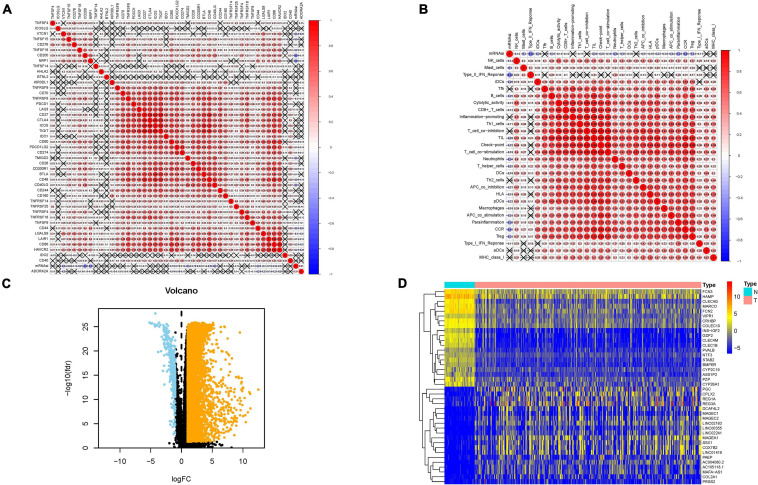
Associations of the stemness index with the tumor immune microenvironment in HCC. **(A)** Broad co-expression correlation among mRNAsi and expression of 47 immune checkpoint blockade genes in HCC. “×” means *p* ≥ 0.05. **(B)** Broad co-expression correlation among mRNAsi and immune infiltration in HCC. “×” means *p* ≥ 0.05. **(C)** Volcano plot was delineated to visualize the differentially expressed genes (DEGs). Orange represents upregulated and blue represents downregulated. **(D)** Heatmap of top 40 DEGs was drawn to reveal different distribution of expression state, where the colors of red to blue represent alterations from high expression to low expression.

Considering that the stemness served as a decisive role in anti-cancer immunity, we speculate that mRNAsi might contribute into diversity of TIME. The results of ESTIMATE exhibited that mRNAsi experienced significantly negative correlation with ESTIMATE score, the presence of immune cells and stromal cells. In the contrary, mRNAsi value was remarkably and positively correlated with tumor purity (Figures 1D–G). Additionally, differences in immune signaling pathways were determined using ssGSEA algorithm under the stratification of stemness index. Interestingly, mRNAsi experienced significant negative correlation with expression of ICB-associated genes (i.e., PDCD1LG2, etc.; Figure 2A). Furthermore, most infiltrating immune cells (i.e., CD8+ T cells, etc.) and immune-related signature (i.e., cytolytic activity, etc.) were significantly and negatively correlated with mRNAsi (Figure 2B), consistent with previous results that mRNAsi were negatively correlated with immunity.

### Identification of Differentially Expressed Genes

Given that mRNAsi value was significantly different between normal and tumor tissues, DEGs analysis was first implemented to elucidate the difference of stemness index from mRNA level. In total, 7,667 genes (7,273 upregulated genes and 394 downregulated genes) were screened as DEGs between HCC tissues and normal samples, which were visualized in the volcano plot (Figure 2C and Supplementary Table 3). The heatmap plot presented the expression distribution of top 40 DEGs (Figure 2D).

### Weighted Gene Co-expression Network Analysis: Identification of the Most Significant Modules and Genes

**FIGURE 3:**
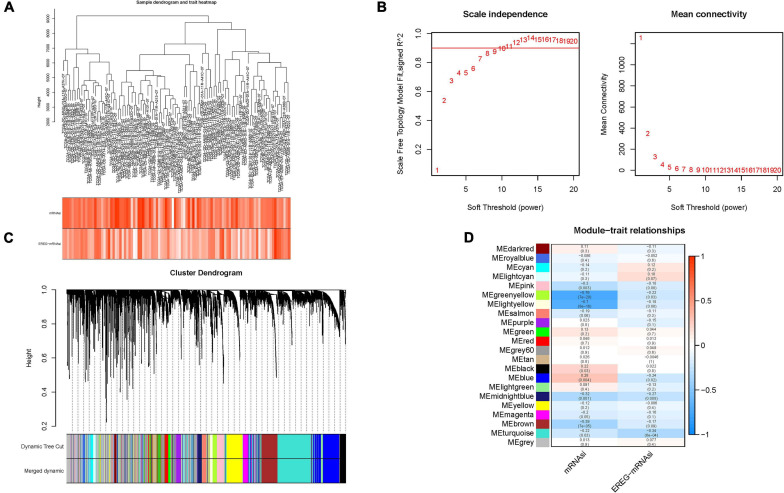
Construction of weighted gene co-expression network of HCC samples. **(A)** Sample dendrogram and clinical-traits heatmap was plotted. **(B)** Selection of the soft threshold made the index of scale-free topologies reach 0.90 and analysis of the average connectivity of 1–20 soft threshold power. **(C)** mRNAsi-related genes with similar expression patterns were merged into the same module using a dynamic tree-cutting algorithm, creating a hierarchical clustering tree. **(D)** Heatmap of the correlations between the modules and mRNAsi as well as epigenetically regulated mRNA expression-based stemness index (EREG-mRNAsi) (traits). Within every square, the number on the top refers to the coefficient between the tumor mutation burden (TMB) level and corresponding module, and the bottom is the *p*-value.

To identify stemness hub genes, WGCNA analysis was performed to construct the co-expression network for mRNA expression data of 7,667 genes together with mRANsi score and EREG-mRNAsi score. Sample dendrogram and stemness-traits heatmap were plotted (Figure 3A). In order to construct the scaleless network, the optimal soft threshold power (β) was set as 10 since it was the first power value when the index of scale-free topologies achieve 0.90 (Figure 3B). Genes with similar expression patterns were introduced into the same module by dynamic tree-cutting algorithm (module size = 50), making a hierarchical clustering tree with modules. Hierarchical clustering analysis was conducted based on weighted correlation, and the clustering results were segmented according to the set criteria (Figure 3C). The parameter was set as 0.25 to merge closely associated modules. Finally, a total of 22 modules were identified (Figure 3D). Then, the MEs indicated that the greenyellow module clearly showed the highest association with stemness (*r* = −0.76, *p* = 7e−20). Therefore, the greenyellow module with 175 genes (Supplementary Table 4) was employed as the module of greatest interest for further analysis. Then expression distribution of these candidate genes in normal and tumor samples was plotted (Supplementary Figure 2A) and that their expression levels were discovered to be significantly dysregulated in tumor samples (Supplementary Figure 2B).

### Gene Ontology and Kyoto Encyclopedia of Genes and Genomes Functional Annotation

To reveal the biological role of mRNAsi hub genes in biological process, KEGG and GO enrichment analyses were performed. For KEGG analysis, the top enriched terms were focal adhesion, human papillomavirus infection, and PI3K−Akt signaling pathway (Supplementary Figure 3A). The results of GO enrichment pathway analysis suggested that hub genes were mainly enriched in extracellular matrix organization, connective tissue development, and extracellular structure organization in biological processes (BP); focal adhesion, cell−substrate junction, and collagen-containing extracellular matrix in cellular components (CC); extracellular matrix structural constituent, cell adhesion molecule binding, and actin binding in molecular function (MF; Supplementary Figures 3B–D and Supplementary Table 5).

### Identification of mRNAsi-Based Prognostic Signature

First, nine of mRNAsi hub genes were significantly correlated with prognosis in the univariate Cox regression analysis with *p*-value < 0.005 (Supplementary Figure 4A).

Next, LASSO Cox regression analysis was performed based on the expression data of the nine genes mentioned above (Supplementary Figure 4B). Finally, an eight-gene (N4BP3, NRGN, ITGB5, FAM110D, LPCAT1, CASQ2, UNC5B, and SLCO2A1) prognostic signature was constructed under the optimal value of λ (Supplementary Figure 4C) to obtain the risk score for HCC samples. The risk score was calculated: risk score = (0.1925 × N4BP3 expression) + (0.0242 × UNC5B expression) + (0.1169 × NRGN expression) + (0.0590 × ITGB5 expression) + (0.2177 × LPCAT1 expression) − (0.0071 × SLCO2A1 expression) − (0.3911 × FAM110D expression) − (0.2451 × CASQ2 expression). Then, HCC samples were divided into the low-risk subgroup (*n* = 183) and high-risk subgroup (*n* = 182) when setting the median value as the cut-off point. All final eight hub genes were highly expressed in tumor tissues than in normal tissues (Supplementary Figures 4D–G). Stratification survival analyses based on the cut-off median expression value of each gene pointed out that high expression of N4BP3 and LPCAT1 was correlated with shorter OS time (Supplementary Figures 4H, I). Interestingly, high expression FAM110D and CASQ2A suggested better prognosis, whereas they were upregulated in tumor samples (Supplementary Figures 4J, K).

### Validation of mRNAsi-Based Prognostic Signature

**FIGURE 4:**
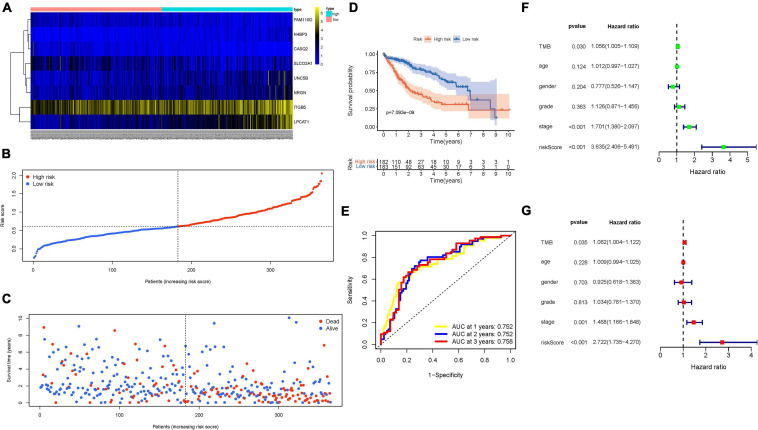
Validation of the prognostic value of risk signature. **(A)** Heatmap presents the expression pattern of three hub genes in each patient, where the colors of yellow to blue represent alterations from high expression to low expression. **(B)** Distribution of multi-genes signature risk score. **(C)** The survival status and interval of HCC patients. **(D)** Kaplan–Meier curve analysis presenting difference of overall survival between the high-risk and low-risk groups. **(E)** Areas under curves (AUCs) of the risk scores for predicting 1-, 2-, and 3-year overall survival time. **(F)** Univariate Cox regression analyses of overall survival. **(G)** Multivariate Cox regression analyses of overall survival.

The distributions of hub gene expression value with corresponding subgroups and patients are delineated in Figure 4A. The allocations of risk score and dot pot of survival status indicated that HCC samples with high risk exhibited poorer prognosis (Figures 4B,C). Moreover, K–M survival analysis demonstrated that low-risk patients had significant higher OS rate (*p* = 7.093e−09; Figure 4D). The predictive value of the signature for OS was validated by time-dependent ROC curves, and the AUC reached 0.752 at 1 year, 0.752 at 2 years, and 0.758 at 3 years (Figure 4E). Univariate and multivariate Cox regression analyses were performed among the available variables to estimate whether the risk score could be an independent prognostic indicator for OS. In single-factor regression analysis, the risk score was discovered to be significantly correlated with OS (HR = 3.635, 95% CI = 2.406–5.491, *p* < 0.001; Figure 4F). After correction for other confounding factors, the risk score still was an independent predictor for OS in the multivariate Cox regression analysis (HR = 2.722, 95% CI = 1.735–4.270, *p* < 0.001; Figure 4G).

The signature was applied to the LIRI-JP cohort to validate the external prognosis predictive performance. Supplementary Figures 5A–C show the distributions of six gene expression patterns, sample survival status, and corresponding risk score in the external validation cohort. Additionally, the survival analysis showed that high-risk HCC patients had a poorer prognosis than low-risk patients (Supplementary Figure 5D; *p* = 1.959e−02). The area under the ROC (AUC) values were more than 0.73 at 1 year in the external validation cohort (Supplementary Figure 5E), which was consistent with our previous results in the training group. However, the AUC was 0.660 at 2 years and 0.662 at 3 years. The good overall prognosis of ICGC-LIRI-JP cohort (5-year OS rate >50%) may weaken prediction accuracy of risk model. Taken together, our results confirmed its external validity among distinct population. Nevertheless, these findings require further validation in more different datasets.

### Clinical Significance of Risk Score

**FIGURE 5:**
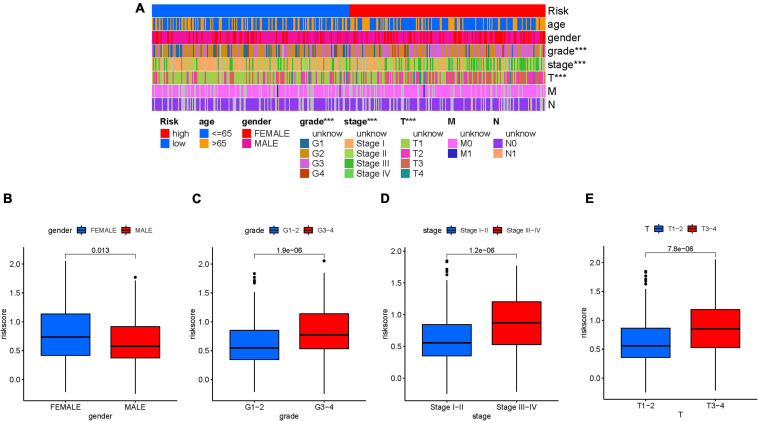
Clinical significance of the prognostic risk signature. **(A)** Heatmap presents the distribution of clinical feature and corresponding risk score in each sample. Comparison of risk score among different subgroups classified by clinical characteristics: gender **(B)**, tumor grade **(C)**, clinical staging **(D)**, and T status **(E)**. The asterisks represented the statistical p value (^∗∗∗^*P* < 0.001).

First, the distribution of clinicopathological feature subtypes in different risk groups was explored and visualized (Figure 5A). For female samples and male samples, risk score presented a higher trend in female samples (Figure 5B). We also observed that patients with late grade also exhibited a significant increase in risk score (Figure 5C). Similarly, risk score was significantly elevated in advanced stage and T3–4 status (Figures 5D, E). Supplementary Figures 6A–D show fraction of subtypes according to gender, pathological grade, clinical stage, and T category in the high-/low-risk group, respectively. These results demonstrated that mRNAsi-based risk score was closely associated with the main clinical characteristics.

### Role of Risk Score in Tumor Immune Microenvironment Context

**FIGURE 6:**
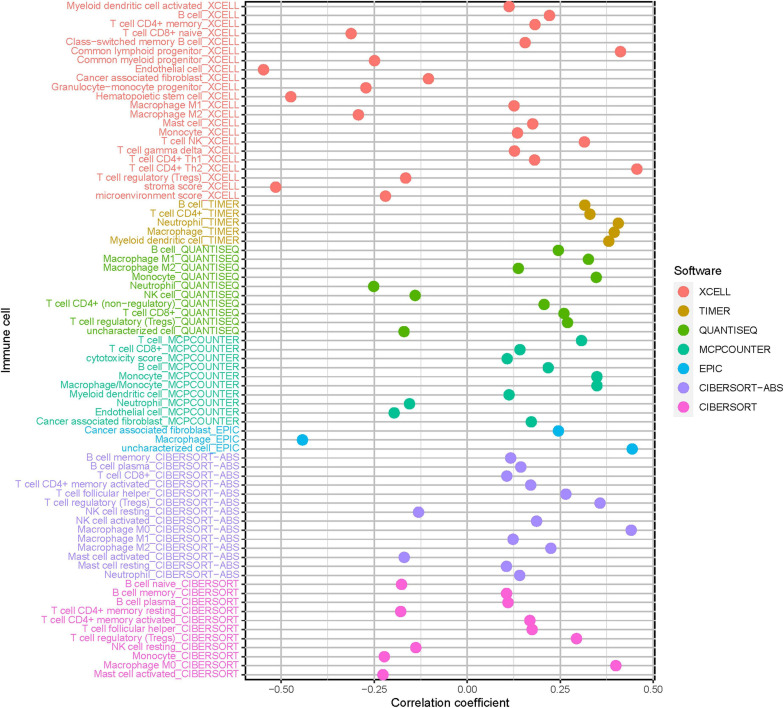
Estimation of tumor-infiltrating cells under risk score in HCC. Patients in the high-risk group were more positively associated with tumor-infiltrating immune cells, as shown by Spearman correlation analysis.

Considering the close association of stemness index with characteristics of TIME, we further explored the potential role of mRNAsi-based signature in diversity and complexity of TIME. The results showed that high-risk score was significantly and negatively correlated with abundance of activated mast cell, neutrophil, and endothelial cell, whereas it was positively related with infiltration of cancer-associated fibroblast, M2 macrophage, resting mast cell, and T-cell regulatory (Tregs; Supplementary Figures 7–9). Furthermore, Spearman correlation analysis was further performed (Figure 6), and the detailed results are provided in Supplementary Table 6. These findings suggested that the low-risk group was characteristic of the presence of antitumor lymphocyte cells, which might enhance anti-tumor effect. In the contrary, the high-risk sample was characterized with infiltration of immunosuppressive immune cell.

### Predicting the Clinical Outcome of Patients to Immunotherapy

**FIGURE 7:**
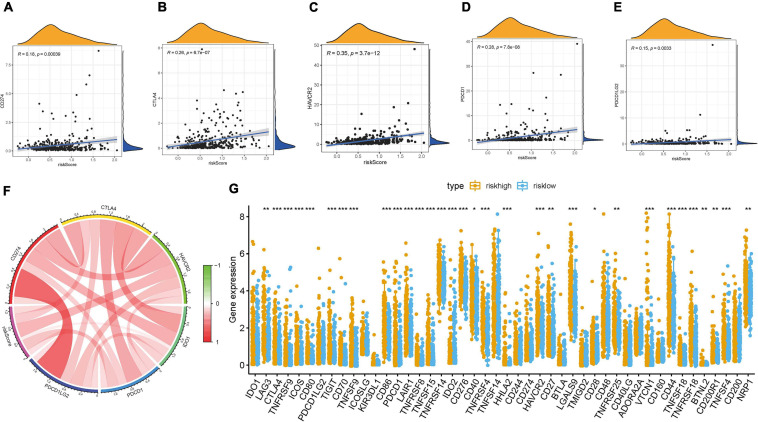
Correlation between prognostic risk signature with hub immune checkpoint genes. **(A)** Correlation between prognostic risk signature and CD274. **(B)** Correlation between prognostic risk signature and CTLA4. **(C)** Correlation between prognostic risk signature and HAVCR2. **(D)** Correlation between prognostic risk signature and PDCD1. **(E)** Correlation between prognostic risk signature and PDCD1LG2. **(F)** Correlation analysis between immune checkpoint inhibitors (CD274, PDCD1, PDCD1LG2, CTLA4, HAVCR2, and IDO1) with prognostic risk signature. **(G)** Differences in expression of 47 immune checkpoint blockade genes between low- and high-risk score groups in the TCGA-LIHC cohort. The asterisks represented the statistical p value (*P < 0.05; **P < 0.01; ***P < 0.001).

Given that the information of immunotherapy treatment was not available in TCGA-LIHC dataset, further analysis was explored for response to immunotherapy. First, we observed that risk score was negatively and significantly correlated with CD274 (*r* = 0.18; *p* = 0.00039), CTLA4 (*r* = 0.26; *p* = 6.7e-07), HAVCR2 (*r* = 0.35; *p* = 3.7e-12), PDCD1 (*r* = 0.28; *p* = 7.8e-08), and PDCD1LG2 (*r* = 0.15; *p* = 0.0033; Figures 7A–E). The correlation of ICB key target (PDCD1, CD274, PDCD1LG2, CTLA-4, HAVCR2, and IDO1) (Kim et al., 2017; Nishino et al., 2017; Zhai et al., 2018) mRNA expression level with risk score was performed (Figure 7F). Additionally, 33 of 47 (i.e., CTLA-4, etc.) ICB-associated targets were significantly upregulated in high-risk samples (Figure 7G). These findings suggested that risk score may act as a determining factor in the regulation of immune response further predicting immunotherapeutic efficacy.

### Gene Mutation in Risk Score

**FIGURE 8:**
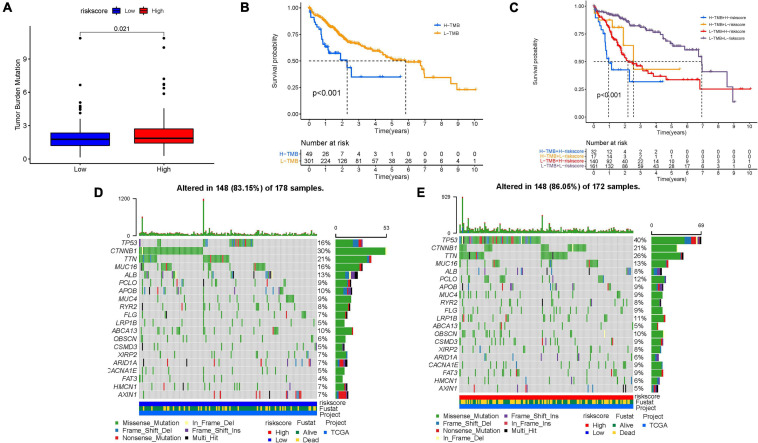
Characteristics of stemness-based risk score in tumor somatic mutation. **(A)** Difference of TMB between patients from the low-/high-risk score subgroups. **(B)** Kaplan–Meier curves for high and low TMB groups. **(C)** Kaplan–Meier curves for patients stratified by both TMB and risk score. The oncoPrint was constructed using low-risk score **(D)** and high-risk score **(E)**.

Increasing evidence has demonstrated an association between the tumor genome somatic mutations and responsiveness to immunotherapy. Consequently, we investigated the distribution patterns of tumor mutation burden (TMB) in different risk score subgroups and discovered that TMB value was higher in the high-risk score subgroup (Figure 8A). Furthermore, no remarkable correlation of risk score with TMB was discovered in HCC (Supplementary Figure 6E). However, the survival curve supported that TMB level significantly and negatively affected OS rate (*p* < 0.001; Figure 8B). The stratified survival curve demonstrated that there was no cross-talk between TMB status and risk score in prognostic predictive value. The different risk score subgroups exhibited remarkable prognostic difference in both high and low TMB value subgroups (*p* < 0.001; Figure 8C). Then, significantly mutated gene (SMG) analysis was further performed in the high-risk score subgroup vs. the low-risk score subgroup. The SMG mutational landscapes presented that CTNNB1 (30 vs. 21%) experienced higher somatic mutation rates in the low-risk score subtype, while TP53 (16 vs. 40%) possessed higher somatic mutation rates in the high-risk score subgroup (Figures 8D, E). These data enabled us to depict the effect of risk score classification on genomic variation more comprehensively, as well as to reveal the potentially complex interaction between individual somatic mutations and stemness.

### Development of Prognostic Nomogram

**FIGURE 9:**
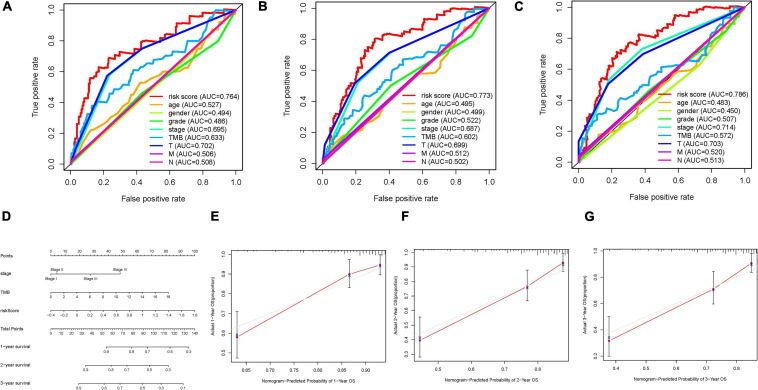
Validation of prognostic efficiency of risk score in HCC. **(A–C)** Areas under curves (AUCs) of the risk scores for predicting 1-, 2-, and 3-year overall survival time with other clinical characteristics. **(D)** Nomogram was assembled by age and risk signature for predicting survival of HCC patients. **(E)** One-year nomogram calibration curves. **(F)** Two-year nomogram calibration curves. **(G)** Three-year nomogram calibration curves.

To corroborate, the risk score was the best predictive indicator; TMB, age, gender, clinical stage, tumor grade, and TNM status were employed as the candidate predictors. These clinical variables were introduced into the AUC analysis for 1-, 2-, and 3-year OS, and risk signature were found to obtain the most AUC value (Figures 9A–C). Then a prognostic nomogram including risk score, TMB, and clinical stage was constructed to predict OS rate quantitatively (Figure 9D). Age, gender, and tumor grade were excluded out of the nomogram because their AUCs were less than 0.6. Calibrated curves were plotted to support great prognostic predictive validity of OS rate in the as-constructed nomogram (Figures 9E–G).

## Discussion

As one of most common and aggressive tumors, HCC was characterized with high morbidity, and patients suffered from poor prognosis (Bray et al., 2018; Forner et al., 2018; Yang et al., 2019). More and more studies reported that such genetic alternation as TP53 mutation, DNA methylation, alternative splicing, and regulation of non-coding RNA played indispensable roles in progression of HCC (Cancer Genome Atlas Research Network, 2017; Xu et al., 2017; Kahles et al., 2018; Wong et al., 2018). Currently, the potential regulatory roles of immune infiltration have attracted increasing attention in anti-tumor treatment, including HCC (Hinshaw and Shevde, 2019; Lu et al., 2019). Cancer immunotherapy, which was designed to employ the effective immune cells to recognize and eliminate cancer cells, has made considerable breakthrough in anti-tumor intervention (Yang, 2015). Clinical studies indicated that the administration of immune checkpoint inhibitors in advanced HCC have achieved encouraging progress; however, only 20% of the patients presented objective response to immunotherapy (Fu et al., 2019). Thus, it is of great significance for predicting prognosis and estimating therapeutic response to optimize clinical benefit and tailored therapeutic strategy.

As a specific subpopulation of tumor cells, CSCs are considered as crucial factors of oncogenesis and therapy resistance (Vidal et al., 2014; Clarke, 2019). Accumulating studies placed emphasis on the regulation of liver CSCs (LCSCs) to elucidate the underlying biological mechanism and promising targets. An independent research reported that LCSCs were relatively resistant to sorafenib and manifested with reduced apoptosis and improved viability (Xin et al., 2013). K19+ HCC cells presented the elevated level of EMT markers together with CSC-like features, suggesting a close correlation of LCSCs with the EMT phenotype (Kawai et al., 2015). The high expression of IL-6 in the LCSC niche was correlated with aggressive recurrence and metastasis in HCC (Wang et al., 2016). Mounting research has supported that LCSCs could reprogram tumor microenvironment into immunosuppressive phenotype through certain extrinsic and intrinsic mechanisms, leading to immune evasion (Dai et al., 2021). However, the comprehensive understanding of LCSCs in prognostic prediction, TIME contexture, and immunotherapy in HCC have not been elucidated fully.

In this context, our focus was on the LCSC-related genes based on an mRNAsi index, as created *via* the OCLR algorithm by Malta et al. (2018). First, the transcriptomic and epigenomic stemness characteristics of HCC were identified based on clinical features. The mRNAsi value was higher in tumor tissues and escalated as the tumor pathological grade elevated, and poorer OS experienced the higher stemness characteristics. With the help of ssGSEA approach and ESTIMATE algorithm, preliminary understanding of the biological role of the stemness features in TIME was established. The lower mRNAsi score was discovered to be significantly associated with abundant immune cell infiltration and higher expression of immunotherapeutic targets, suggesting that low mRNAsi tumor might be more suitable for immunotherapy.

Taking advantage of DEG analysis followed by WGCNA co-express network, 175 mRNAsi-related hub genes were recognized, 9 of which possessed significant prognostic value. The results of subsequent enrichment pathway analysis presented that hub genes were mainly enriched in extracellular matrix structural related pathways, focal adhesion, and PI3K−Akt signaling pathway. By using LASSO Cox regression analysis, candidate hub genes were further determined, and final prognostic signature, including N4BP3, NRGN, ITGB5, FAM110D, LPCAT1, CASQ2, PLN, UNC5B, and SLCO2A1, was constructed. All of these genes were dysregulated in tumor tissues, four of which could identify prognosis differences in HCC. Interestingly, FAM110D and CASQ2 were overexpressed in tumor samples, whereas a higher level of them represented longer OS time, indicating such underlying mechanism as stemness characteristic might lie in their prognostic value.

To validate prognostic accuracy of as-constructed model, survival analysis and ROC curve were performed. Furthermore, risk score was demonstrated to serve as an independent prognostic indicator under both univariable and multivariable Cox regression analysis. Further validation was performed using an external dataset (ICGC-LIRI-JP cohort). Furthermore, risk score was discovered to be associated with the main clinical variables (gender, tumor grade, clinical stage, and T status).

Considering risk signature derived from stemness index, which was significantly correlated with anti-tumor immunity, the potential role of risk score in complexity of TIME and immunotherapeutic effect was further investigated. The results pointed out that risk score was positively related with activated immune cell (i.e., cancer-associated fibroblast, M2 macrophage, etc.), implying that patients with a high-risk score was characteristic of immunosuppressive phenotype. In consistent with survival analysis, high-risk score patients exhibited a matching survival inferiority. In addition, risk score was significantly and positively correlated with the immunotherapeutic hub targets (i.e., PDCD1, CTLA4, etc.), suggesting that samples with a high-risk score might be more affected by ICB pathways, then inhibited anti-tumor immune activation, and deteriorated prognosis accordingly. These findings indicated that tumors with a high-risk score might obtain clinical benefit from immunotherapy. In the absence of immunotherapy data in HCC cohort, it was unable to further explore the correlation of risk score with response to immunotherapy.

Exploration of the somatic mutation underlying progression of tumor functioned as indispensable foundation for diagnostic practice, therapeutic intervention, and prognostic prediction. In our study, the TP53 mutation rates were revealed to be markedly augmented in the high-risk score subtype, while the mutation rate of the SMGs of CTNNB1 was increased in the patients with low-risk score. Previous researches indicated that TP53, of which mutation leads to the downregulation of the immunotherapeutic response in HCC, is one of the most frequently mutated genes in multiple cancer types (Long et al., 2019). According to existing papers, mutation of CTNNB1, remarkably characterizing the immune-excluded phenotype, could serve as a novel indicator for prediction of immunotherapeutic resistance in HCC (Pinyol et al., 2019). The distribution differences of mRNAsi-related mutated driver genes were significantly correlated with the anti-tumor immunity, highlighting the complicated interaction of stemness characteristics with somatic mutation contributing to tumor immunogenomic regulation. A subsequent stratified survival curve demonstrated that the risk score had a prognostic predictive capability that was independent of the TMB, suggesting that somatic mutation and stemness represent different aspects of immunobiology.

Considering the multiple and complex factors of clinical outcome between individual tumors, it was of great urgency to define the individual patient prognosis quantitatively. As such, comprehensive prognostic scoring scheme (TMB-clinical-risk nomogram) were established to quantify the distinct OS probability, further contributing to clinical practice.

Compared with published articles that investigated the stemness characteristics in HCC, it was worthy to mention that there were some superiorities in this work. First, seven novel and reliable algorithms (XCELL, TIMER, QUANTISEQ, MCPcounter, EPIC, CIBERSORT, and CIBERSORT-ABS) were performed to the potential roles of stemness-based risk in the formation of TIME diversity and complexity. In addition, the underlying interaction of stemness-based risk with immunotherapy was preliminarily explored, and the synergistic effect of stemness-based risk with TMB was uncovered. Moreover, a robust and promising prognostic TMB-clinical-risk nomogram with encouraging potential for clinical practice was constructed to predict individual sample clinical outcome quantitatively.

## Conclusion

In conclusion, the comprehensive analysis of mRNAsi hub genes in the context of TIME will facilitate understanding stemness characteristics from biological standpoint and contribute to the tailored immunotherapeutic administration. Notwithstanding, these results required further experimental and more clinical exploration focusing on oncogenesis and progression and the underlying mechanism of regulation based on stemness in HCC.

## Data Availability Statement

The original contributions presented in the study are included in the article/Supplementary Material, further inquiries can be directed to the corresponding author/s.

## Author Contributions

WH designed the overall study and revised the manuscript. QX performed the public data interpretation and drafted the manuscript. HX and SC participated in the data collection. QX and HX contributed to the data analysis. All authors read and approved the final manuscript.

## Conflict of Interest

The authors declare that the research was conducted in the absence of any commercial or financial relationships that could be construed as a potential conflict of interest.

## Publisher’s Note

All claims expressed in this article are solely those of the authors and do not necessarily represent those of their affiliated organizations, or those of the publisher, the editors and the reviewers. Any product that may be evaluated in this article, or claim that may be made by its manufacturer, is not guaranteed or endorsed by the publisher.

## Footnotes

1
https://portal.gdc.cancer.gov/

2
http://asia.ensembl.org/index.html

3
https://bioinformaticsfmrp.github.io/PanCanStem_Web/

4
https://dcc.icgc.org

## Abbreviations

AUC: area under the curve
BP: biological processes
CC: cellular components
CD274: also known as PD-L1
CI: confidence interval
CTLA-4: cytotoxic T-lymphocyte -associated antigen 4
CSCs: cancer stem cells
DCs: dendritic cells
DEG: differentially expressed genes
DEL: deletion
EREG-mRNAsi: epigenetically regulated mRNA expression-based stemness index
ESTIMATE: Estimation of Stromal and Immune Cells in Malignant Tumors using Expression Data
FC: fold change
FDR: false discovery rate
GO: Gene Ontology
GS: gene significance
HCC: hepatocellular carcinoma
HR: hazard ratio
ICB: immune checkpoint blockade
IDO1: indoleamine 2,3-dioxygenase 1
INS: insertion
KEGG: Kyoto Encyclopedia of Genes and Genomes
K–M: Kaplan–Meier
K–W: Kruskal–Wallis
LASSO: least absolute shrinkage and selection operator
LCSCs: liver cancer stem cells
MAF: Mutation Annotation Format
MEs: module eigengenes
MF: molecular function
mRNAsi: mRNA expression-based stemness index
OCLR: one-class logistic regression
OS: overall survival
PDCD1: also known as PD-1
PDCD1LG2: also known as PD-L2
ROC: receiver operating characteristic
SNP: single-nucleotide polymorphism
ssGSEA: single-sample gene-set enrichment analysis
TCGA: The Cancer Genome Atlas
TICs: tumor-infiltrating immune cells
TILs: tumor-infiltrating lymphocytes
TIM-3: T-cell immunoglobulin and mucin-domain containing-3
HAVCR2: also known as TIM-3
TIME: tumor immune microenvironment
TMB: tumor mutation burden
TNM: tumor–node–metastasis
TOM: topological overlap matrix
WGCNA: weighted gene co-expression network analysis.
