# Supplementary material for: Immunological Value of Prognostic Signature Based on Cancer Stem Cell Characteristics in Hepatocellular Carcinoma
Source: Front Cell Dev Biol. 2021 Aug 2;9:710207. doi: 10.3389/fcell.2021.710207 (PMC8365341; doi:10.3389/fcell.2021.710207)
Supplement: Supplementary file 1 [file Data_Sheet_1.DOCX]

**Supplementary Figure Legends:**

**Figure S1:** Clinical characteristics and molecular features correlated with the epigenetically regulated mRNAsi (EREG-mRNAsi) in HCC (A) Differences in EREG-mRNAsi between normal (50 samples) and tumor (374 samples) tissues. (B) Kaplan-Meier curves show that the low EREG-mRNAsi subgroup had greater mortality than does the high EREG-mRNAsi subgroup. (C) Boxplots of EREG-mRNAsi value in individual samples stratified by tumor grade.

**Figure S2:** (A) Heatmap of mRNAsi-related genes was drawn to reveal different distribution of expression state, where the colors of red to blue represented alterations from high expression to low expression. (B) Comparison of expression level of mRNAsi-related genes between tumor samples and normal tissues.

**Figure S3: Functional annotation of mRNAsi-related genes.** (A) KEGG enrichment analysis of mRNAsi-related genes. (B-C) Gene Ontology (GO) enrichment analysis of mRNAsi -related genes: biological processes (BP), cellular components (CC) and molecular function (MF).

**Figure S4.** Identification of the candidate stemness-related genes with prognostic value. (A) Forest plots showing the results of the univariate Cox regression analysis between gene expression and OS. (B) LASSO coefficient profiles of the expression of 26 candidate genes. (C) Selection of the penalty parameter (λ) in the LASSO model via 10-fold cross-validation. The dotted vertical lines are plotted at the optimal values following the minimum criteria (left) and “one standard error” criteria (right). (D) LPCAT1 expression level of mRNA in TCGA cohort. (P=1.782e−16). (E) N4BP3 expression level of mRNA in TCGA cohort. (P=1.127e−20). (F) CASQ2 expression level of mRNA in TCGA cohort. (P=7.034e−11). (G) FAM110D expression level of mRNA in TCGA cohort. (P=3.366e−10). Survival analysis between high- and low- expression groups of hub genes. (H) LPCAT1, (I) N4BP3, (J) CASQ2, and (K) FAM110D.

**Figure S5.** Validation of the prognostic value of risk signature. (A) Heatmap presents the expression pattern of three hub genes in each patient, where the colors of yellow to blue represented alterations from high expression to low expression. (B) Distribution of multi-genes signature risk score. (C) The survival status and interval of HCC patients. (D) Kaplan–Meier curve analysis presenting difference of overall survival between the high-risk and low-risk groups. (E) Areas under curves (AUCs) of the risk scores for predicting 1-, 2-, and 3-year overall survival time.

**Figure S6: Rate of clinical variables subtypes in high or low risk score groups.** (A) Gender, (B) WHO grade, (C) clinical stage, and (D) T status. (E) Scatterplots depicting the correlation between risk scores and TMB value.

**Figure S7-S9: The representative results of the evaluation of tumor infiltrating immune cells with risk signature.**
