# Supplementary figures and images for: Immunological Value of Prognostic Signature Based on Cancer Stem Cell Characteristics in Hepatocellular Carcinoma
Source: Front Cell Dev Biol. 2021 Aug 2;9:710207. doi: 10.3389/fcell.2021.710207 (PMC8365341; doi:10.3389/fcell.2021.710207)

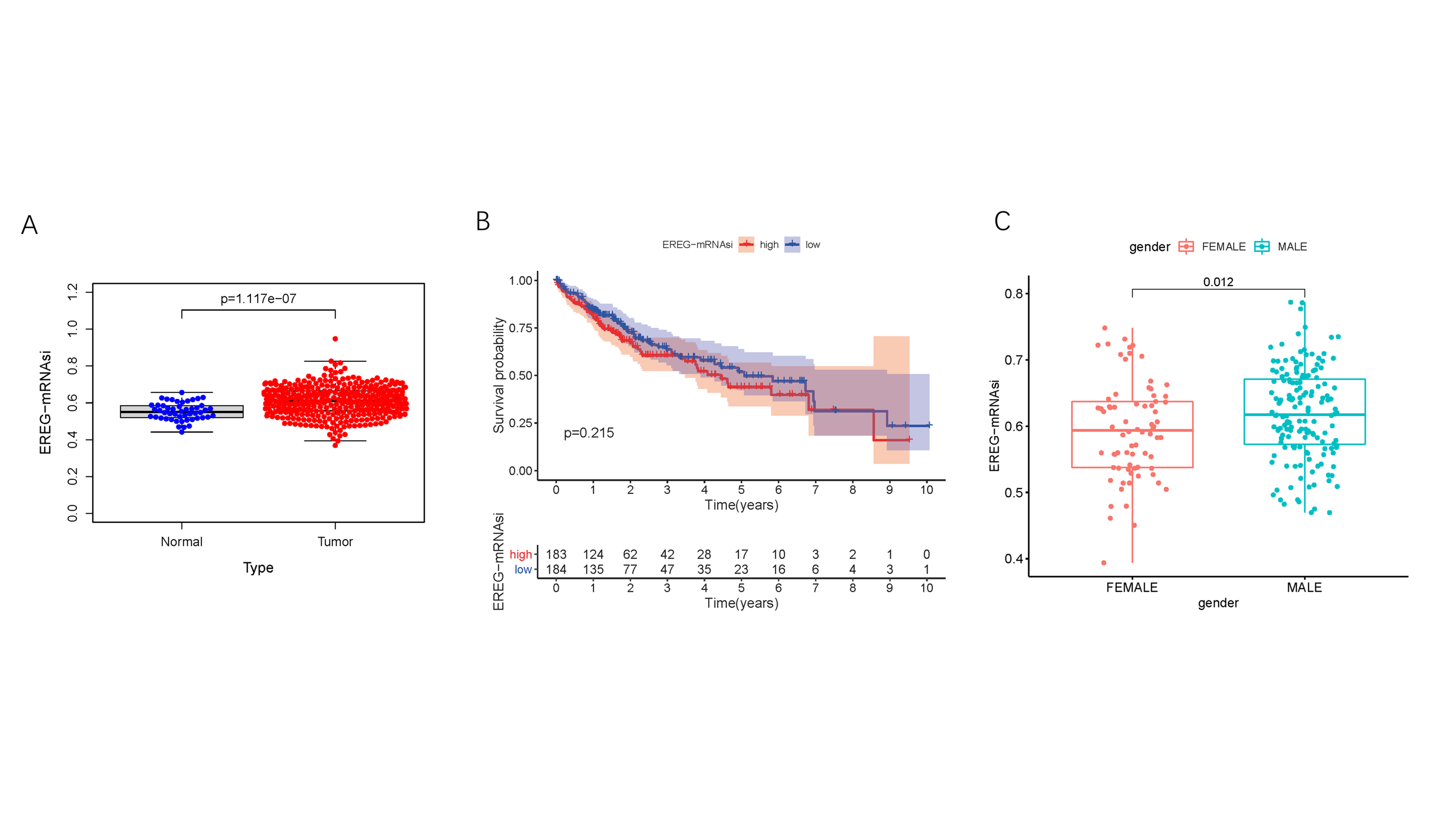

Supplement: Supplementary file 3 [file Image_1.TIF]

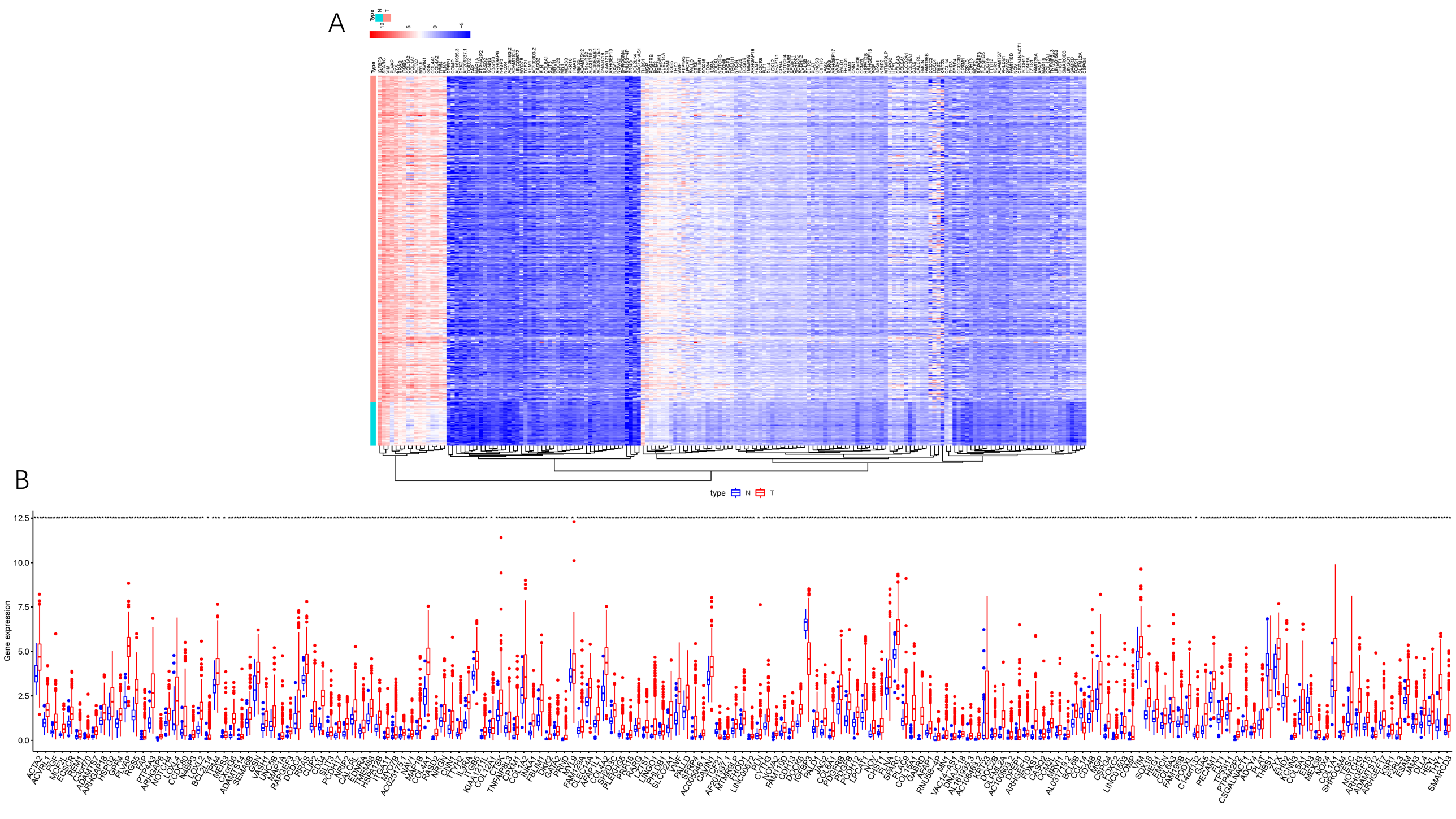

Supplement: Supplementary file 4 [file Image_2.TIF]

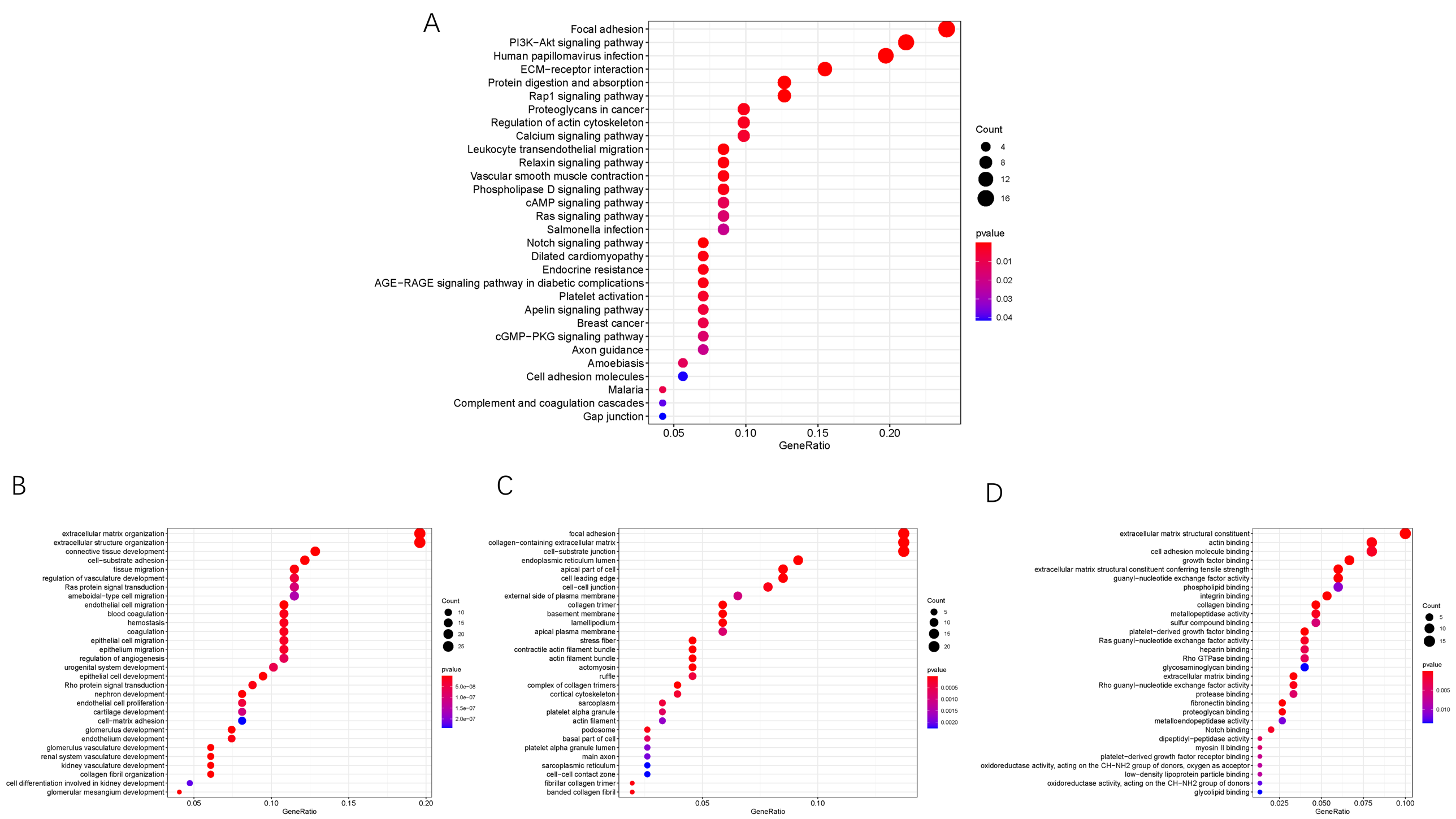

Supplement: Supplementary file 5 [file Image_3.TIF]

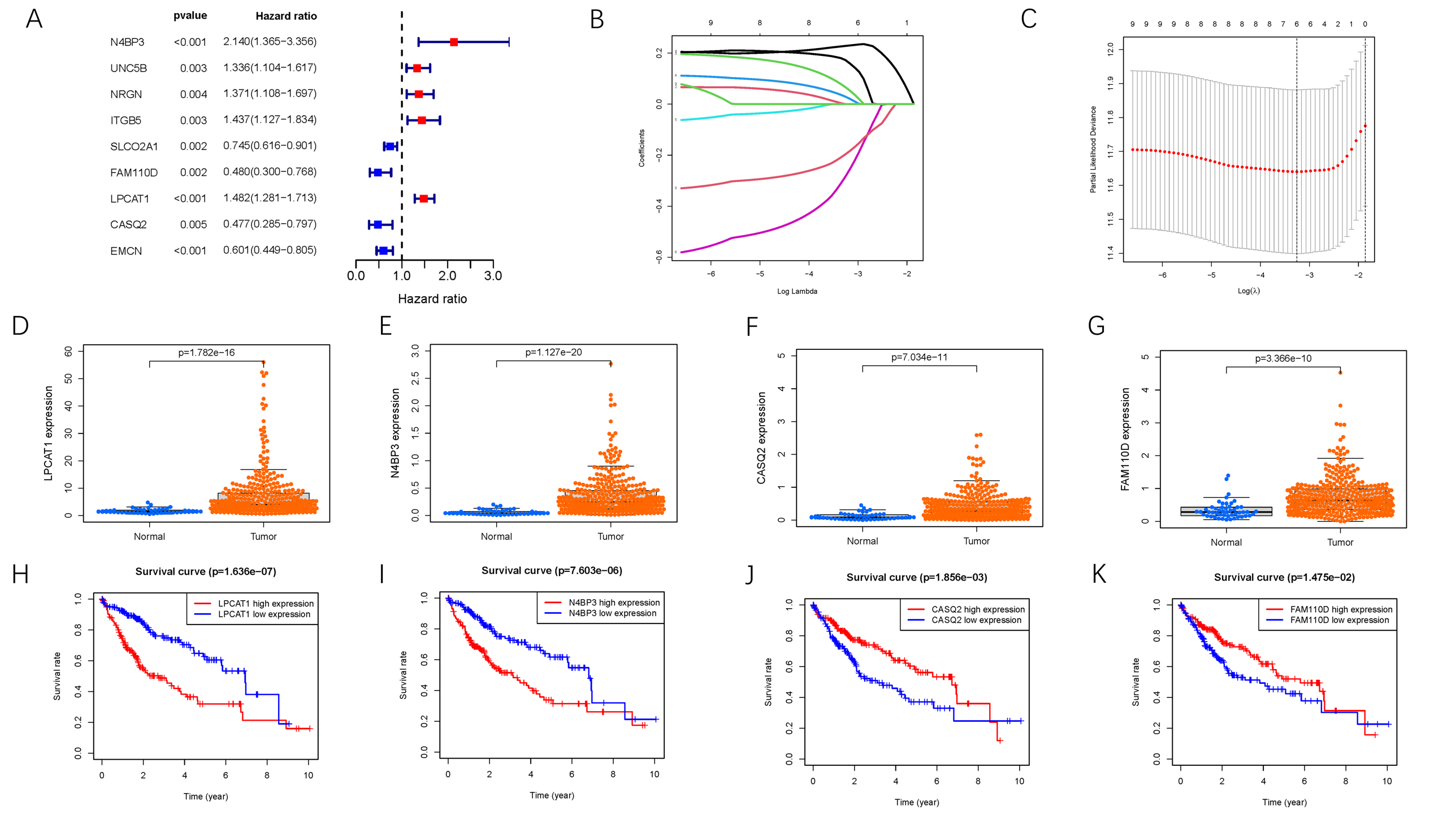

Supplement: Supplementary file 6 [file Image_4.TIF]

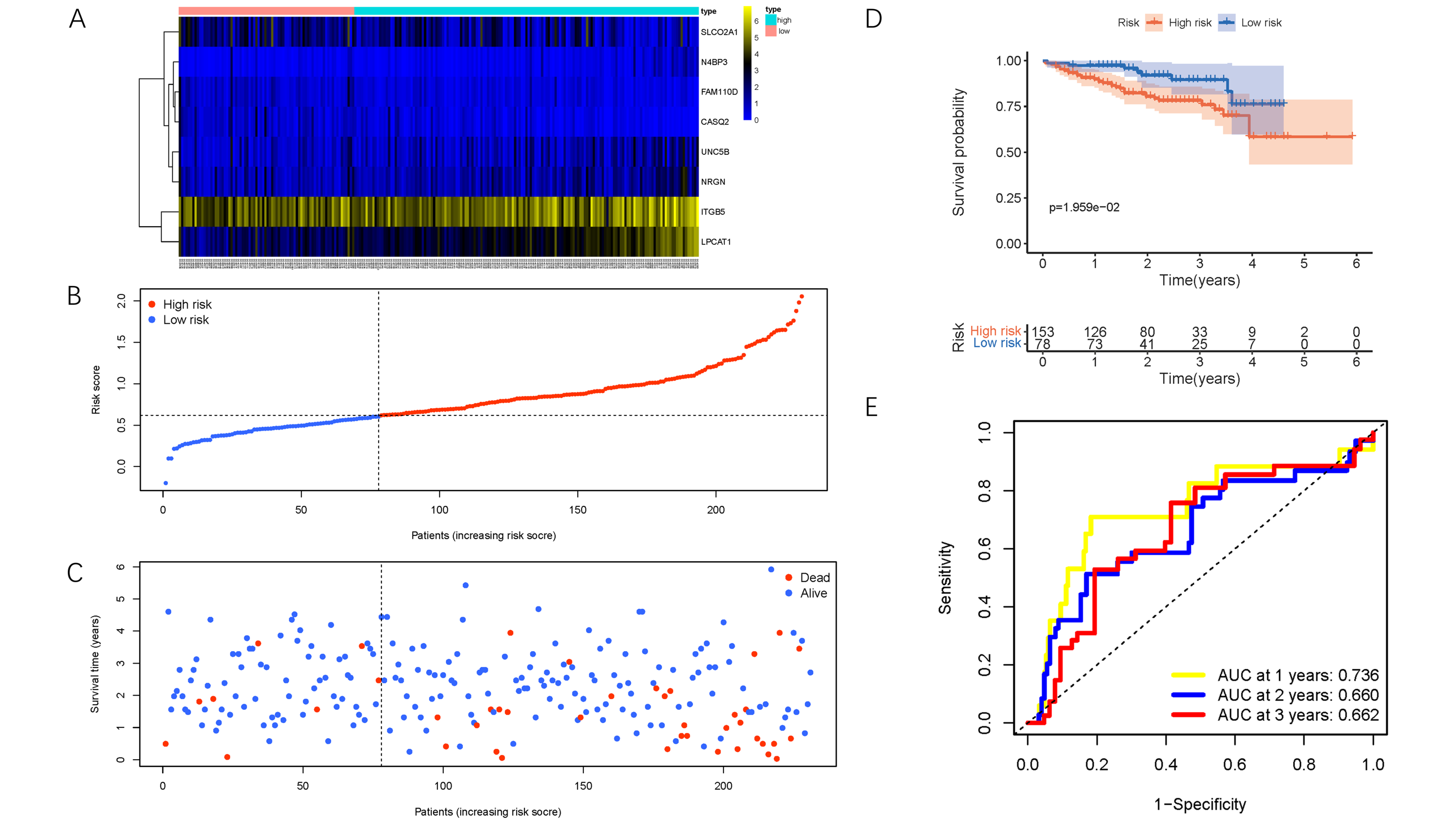

Supplement: Supplementary file 7 [file Image_5.TIF]

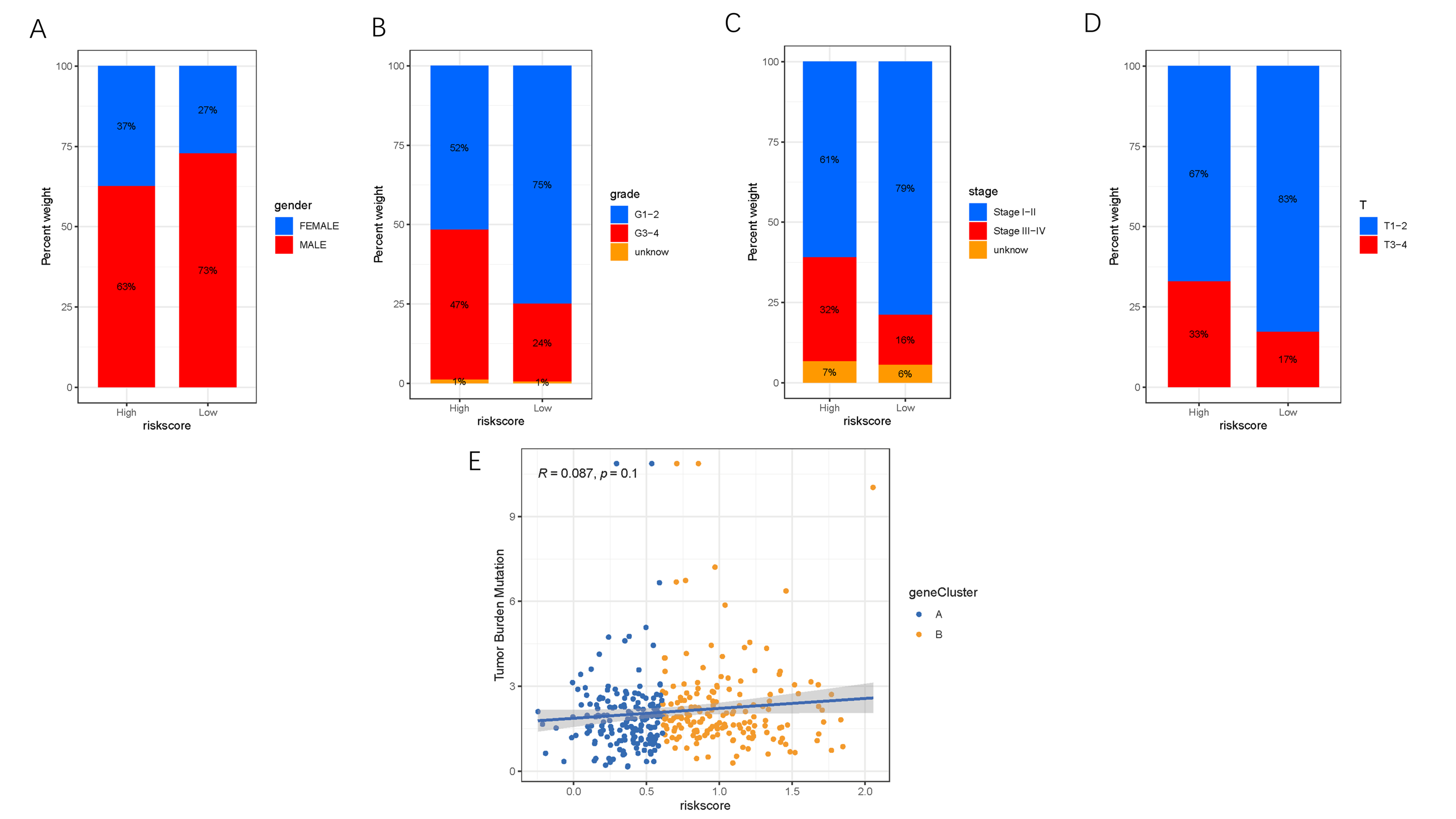

Supplement: Supplementary file 8 [file Image_6.TIF]

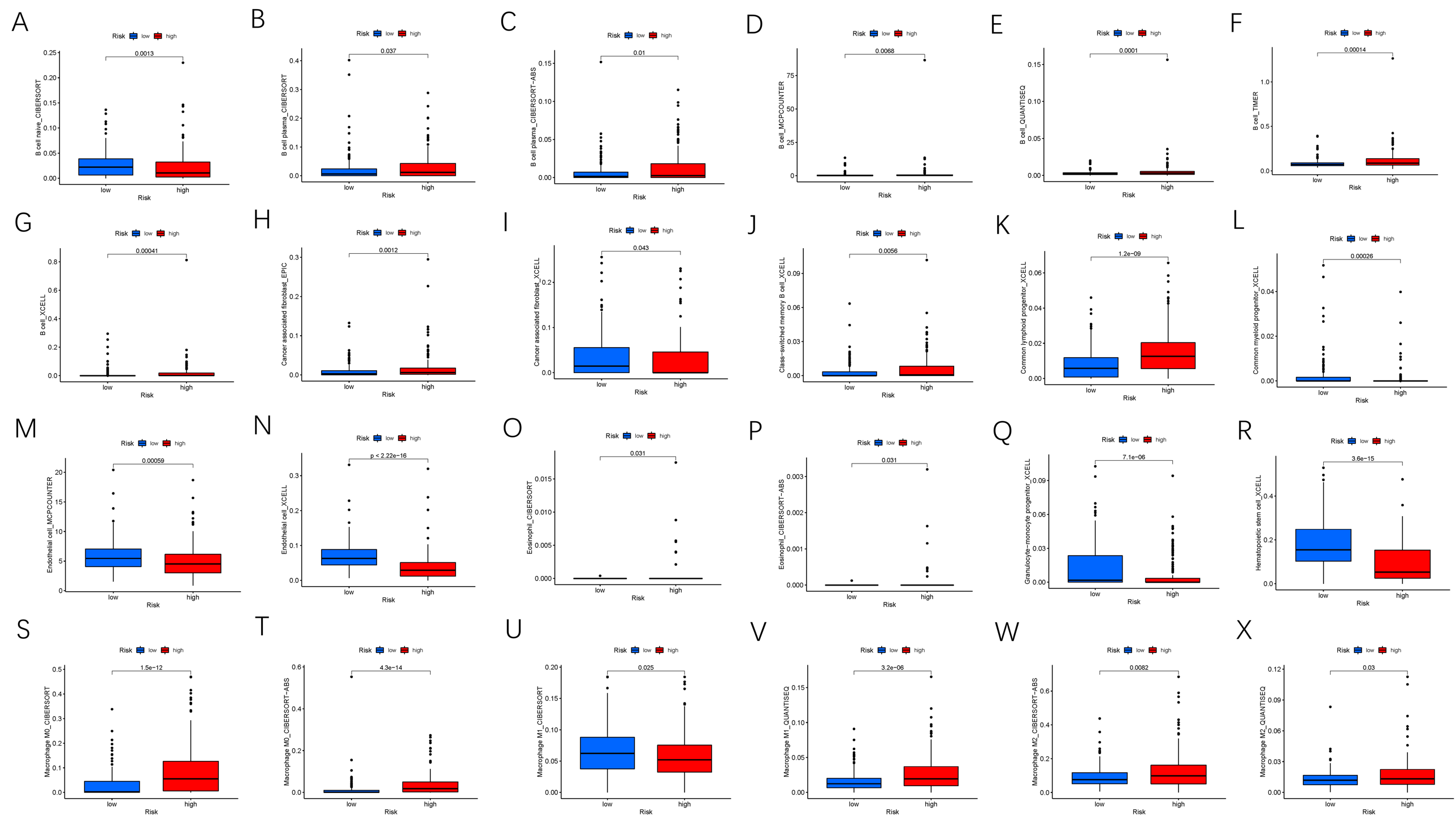

Supplement: Supplementary file 9 [file Image_7.TIF]

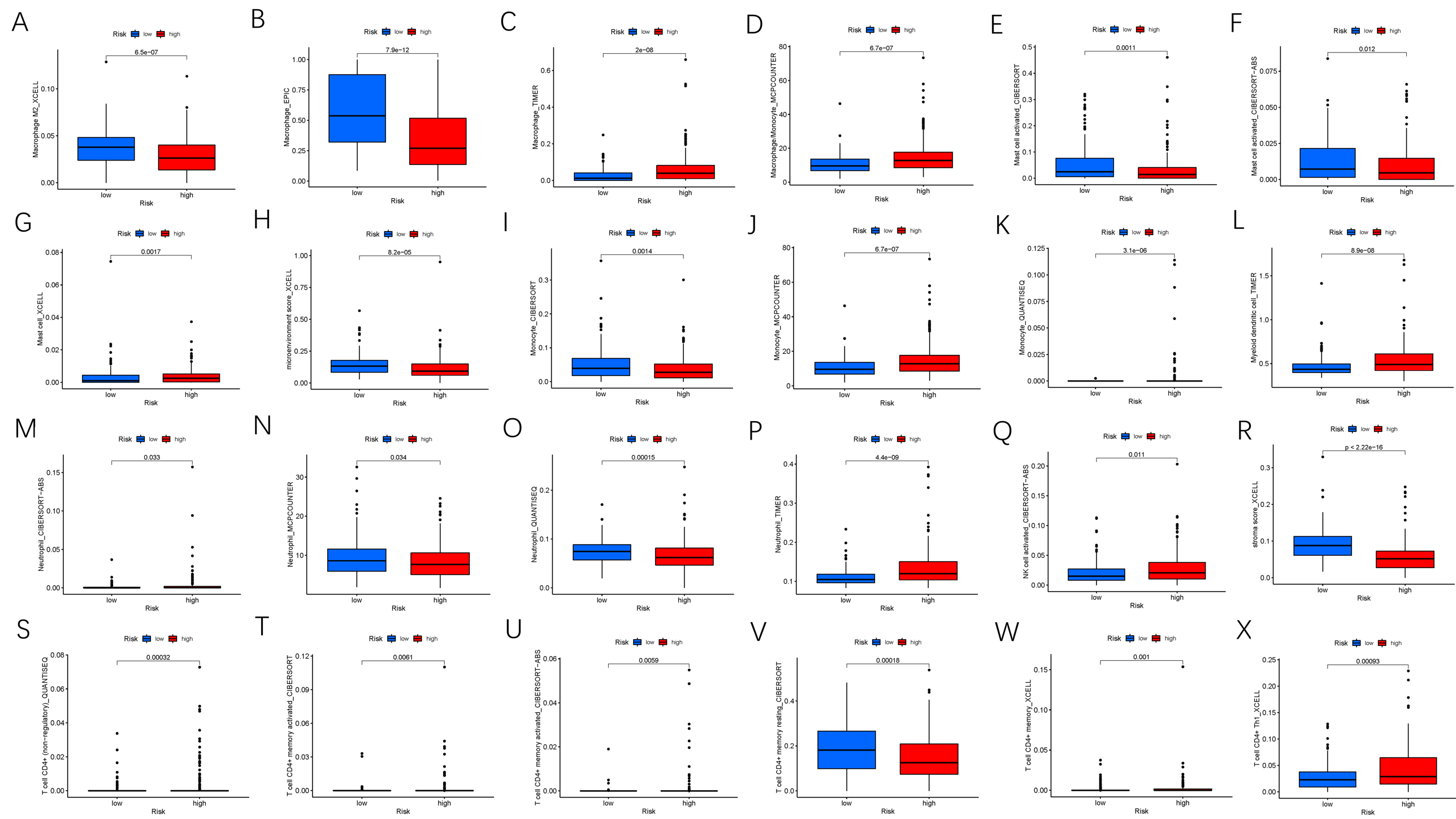

Supplement: Supplementary file 10 [file Image_8.TIF]

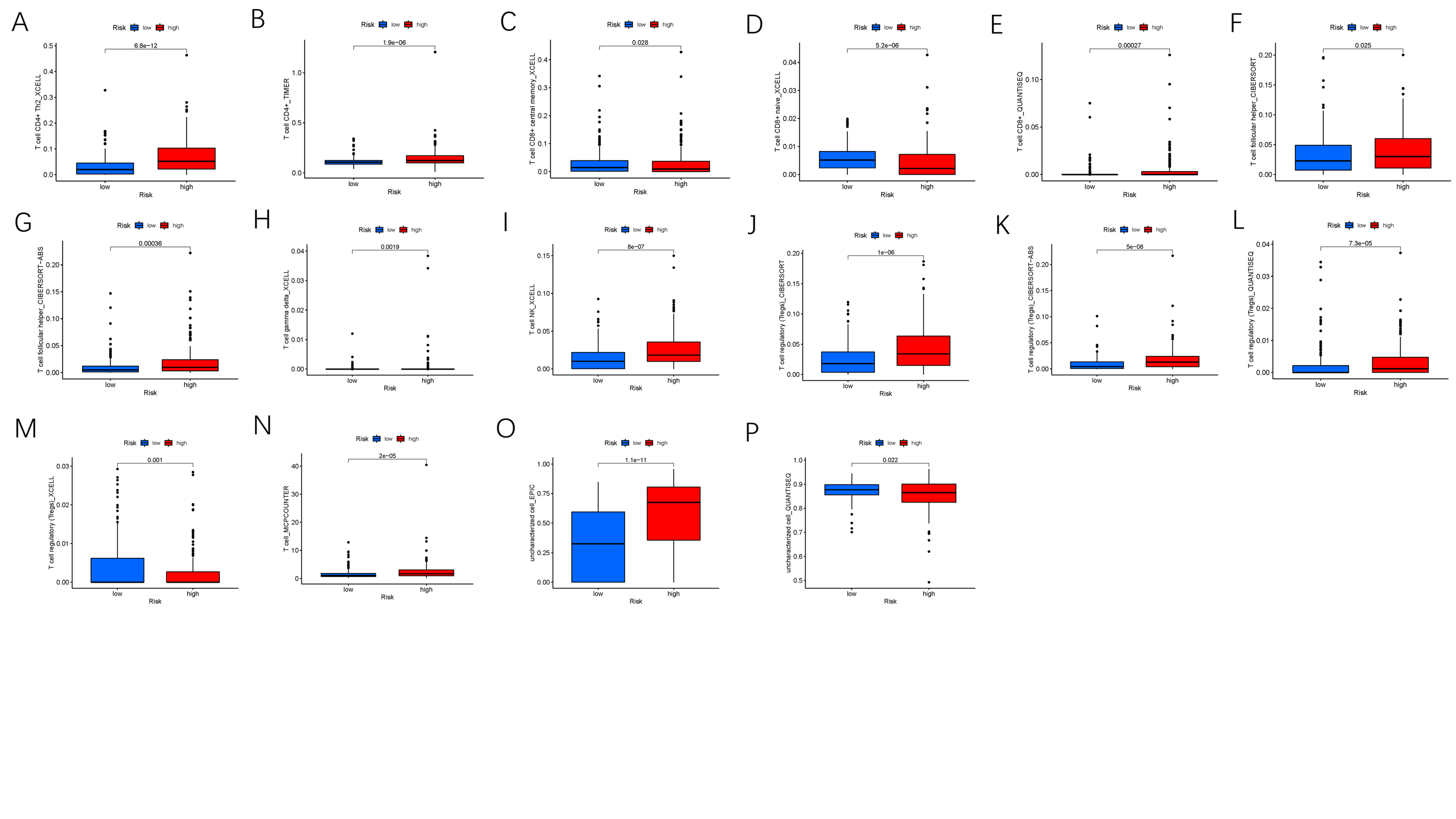

Supplement: Supplementary file 11 [file Image_9.TIF]
